# *In Vitro* and *In Vivo* Evaluation of Oxatomide ***β***-Cyclodextrin Inclusion Complex

**DOI:** 10.1155/2013/629593

**Published:** 2012-12-06

**Authors:** Fahima M. Hashem, Mohamed Mostafa, Mahmoud Shaker, Mohamed Nasr

**Affiliations:** ^1^Department of Pharmaceutics and Industrial Pharmacy, Faculty of Pharmacy, Helwan University, Cairo 11790, Egypt; ^2^Department of Pharmaceutics, Faculty of Pharmacy, Suez Canal University, Ismailia 41522, Egypt

## Abstract

The objective of this study was to evaluate the influence of oxatomide *β*-cyclodextrin inclusion complex on the physicochemical properties and bioavailability of the drug. Oxatomide *β*-cyclodextrin solid complex was prepared with equimolar ratio of both oxatomide and *β*-cyclodextrin in presence or absence of water soluble polymers using different techniques. The coevaporated complex prepared in presence of PVP-K15 showed a prompt drug release and significantly increased % dissolution efficiency (*P* < 0.05) compared to the pure oxatomide. Moreover, the results of bioavailability evaluation of this complex in rabbits compared to commercial drug product indicated a 73.15% increase in the oral bioavailability of oxatomide. In conclusion, inclusion complex of oxatomide with *β*-cyclodextrin prepared by coevaporation in presence of PVP-K15 not only results in an enhancement of the oxatomide dissolution rate but also improves the bioavailability of oxatomide.

## 1. Introduction 

Oxatomide is an antihistaminic drug that has been reported to have applications in the treatment of a number of different types of allergic and other hypersensitivity reactions. These include but are not restricted to the symptomatic treatment of allergic rhinitis and chronic urticaria, the classical nasal and ocular symptoms associated with hay fever, the reduction of the severity of the erythema, and pruritus in cases of chronic urticaria [1, 2]. Although oxatomide has been extensively investigated and approved for the use in the treatment of a broad range of diseases, it still possesses bioavailability limitations and poor dissolution properties that restrict its full clinical use [1]. 

One of the major current challenges of the pharmaceutical industry is related to strategies that improve the water solubility of drugs [3, 4]. Low solubility can cause low bioavailability or give rise to large fluctuations in the fraction absorbed in humans that can often not be compensated by a high permeability. Furthermore, low solubility may be associated with stability problems and difficulties in developing an acceptable formulation [5]. By improving the drug release profile of these drugs, it is possible to enhance their bioavailability and reduce side effects [6–9]. 

Cyclodextrins have been utilized extensively in pharmaceutical formulations to enhance oral bioavailability [10]. Cyclodextrins are a family of cyclic oligosaccharides that are composed of *α*-1, 4-linked glucopyranose subunits. These macrocyclic carbohydrates with lipophilic central cavities and hydrophilic outer surfaces can form complexes with and solubilize many normally water-insoluble compounds. The lipophilic cavity of cyclodextrin molecules provides a microenvironment into which appropriately sized nonpolar moieties can enter to form inclusion complexes [11]. Cyclodextrins are of three types: *α*-cyclodextrin, *β*-cyclodextrin, and *γ*-cyclodextrin. Currently, *β*-cyclodextrin is the most common, the most accessible, the lowest-priced, and generally the most useful cyclodextrin in pharmaceutical formulations [12, 13]. In addition, a drug-*β*-cyclodextrin solid inclusion complex is convenient for oral administration [14].

The purpose of study was to improve oxatomide water solubility by preparation of inclusion complex with *β*-cyclodextrin and evaluate the influence of complex formation on the physicochemical properties and bioavailability of the drug.

## 2. Materials and Methods

### 2.1. Materials

Oxatomide was kindly donated by Janssen Pharmaceutica, Germany. *β*-cyclodextrin hydrate was purchased from Acros Organics, New Jersey, USA. Hydroxy propyl methyl cellulose (HPMC) and polyvinyl pyrrolidone K15 (PVP-K15) Fluka, Chemie, USA. Oxatomide tablets (Tinset 30 mg) were purchased from local pharmacy. All other reagents used in this study were of analytical grade. All chemicals were used as received without any further purification.

### 2.2. Methods

#### 2.2.1. Construction of the Oxatomide-Cyclodextrin Phase Solubility Diagrams

An excess of oxatomide was added to screw-capped vials containing aqueous solutions of various concentrations of *β*-cyclodextrin. The vials were mechanically shaken for 48 hrs at room temperature. After 2 days, aliquots were withdrawn and filtered using 0.45 *μ*m pore size filter. The filtrate was suitably diluted and analyzed spectrophotometrically at the wavelength of 226 nm. The same procedure was repeated with *β* cyclodextrin aqueous solution containing either 0.25% w/v PVP-K15 or 0.1% w/v HPMC [15]. 

#### 2.2.2. Preparation of Oxatomide-Cyclodextrin Solid Complexes

Oxatomide *β*-cyclodextrin solid complex was prepared with equimolar ratio of both oxatomide and *β*-cyclodextrin by using five different techniques as follows.

*Physical Mixing Technique. *The technique simply relies on the idea that both oxatomide and *β*-cyclodextrin were properly blended together in a ceramic mortar using pestle. 

*Kneading Technique. *Into a ceramic mortar, *β*-cyclodextrin was wetted with a few drops of distilled water and properly kneaded. Then, oxatomide was added slowly, and the combined mixture was kneaded with the addition of few drops of distilled water. The dough mass was pressed and stretched with the hand fingers, folded over, and rotated through 90°, repeatedly. This process was continued for additional 15 min until the dough is elastic and smooth and then the obtained dough mass was left to dry under fume hood at room temperature for 24 hrs. 

*Coevaporation Technique. *Coevaporation is another technique for formation of solid oxatomide *β*-cyclodextrin complex. Simply it consists of the simultaneous deposition of the oxatomide and *β*-cyclodextrin under vacuum conditions. This is simply done by dissolving weighed amount of oxatomide in 10 mL of 60% methanol solution in a glass beaker. In another glass beaker, *β*-cyclodextrin was dissolved in 50 mL distilled water. The contents of the two beakers are then mixed together, and the obtained solution is put in sonicator for 25 min at room temperature to obtain a clear solution. The resultant solution is dried by being subjected to evaporation using Büchi Rotavapor (R200V800, Büchi Labortechnik, Switzerland) at 70°C under vacuum (55 mbar). 

*Spray-Drying Technique. *Spray drying was performed in a Büchi mini spray drier (B 290, Büchi Labortechnik, Switzerland). This is simply done by dissolving weighed amount of oxatomide in 10 mL 60% methanol solution, in 100 mL glass beaker. In another 100 mL glass beaker *β*-cyclodextrin was dissolved in 50 mL distilled water. The contents of the two beakers were then mixed together and sonicated at room temperature for 25 min to obtain and ensure a clear solution. The obtained solution is dried by spray drying under purified nitrogen gas. The inlet adjusted to a flow rate of 880 mL/h and at temperature 120°C. The nitrogen gas adapted  to a flow rate of 357 NL/h u, and outlet temperature was 85°C. 

*Freeze-Drying Technique. *Freeze-drying process was typically carried out in Heto Power Dry LL 3000 freeze dryer (Thermo Electron Corporation, Czech Republic) at −50°C, under vacuum (0.128 mbar). Briefly, in a 100 mL glass beaker, oxatomide was dissolved in 10 mL 60% methanol solution. In another 100 mL glass beaker, *β*-cyclodextrin was dissolved in 50 mL distilled water. The contents of the two beakers are then mixed together, and the obtained solution is put in sonicator at room temperature for 25 min to ensure that a clear solution is obtained. The resultant solution is then frozen by placing at −30°C freezer for at least 24 hrs to ensure that the solution was completely frozen. Then, frozen solution was kept for 24 hrs into the vacuum chamber at −50°C where the freeze-drying process takes place.

#### 2.2.3. Preparation of Oxatomide-Cyclodextrin Solid Complex in Presence of Water Soluble Polymer

Oxatomide *β*-cyclodextrin solid complexes were prepared with a molar ratio of 1 : 1 in addition to 21% of water soluble polymer such as PVP-K15, HPMC, or PEG 6000 using coevaporation, spray-drying, and freeze-drying techniques. 

#### 2.2.4. Characterization of Oxatomide-Cyclodextrin Complex

The oxatomide-*β*-cyclodextrin complex obtained from each technique is subjected to different chemical and thermal characterizations as follows. 

*Infrared (IR) Spectroscopy. *IR spectra of oxatomide, *β*-cyclodextrin, and the prepared oxatomide *β*-cyclodextrin solid complexes were obtained at room temperature using an infrared spectrometer (IR100/IR200, Thermo Nicolet Corp., USA). Samples were prepared in KBr disks by means of a hydrostatic press. The scanning range was 400 to 4000 cm^−1^, and the resolution was 4 cm^−1^. 

*Differential Scanning Calorimetry (DSC). *The thermal properties of oxatomide, *β*-cyclodextrin, and the prepared oxatomide *β*-cyclodextrin solid complexes were characterized using Shimadzu Differential Scanning Calorimeter, DSC60, Japan. The measurements were carried out at a heating rate of 10°C/min. In order to provide the same thermal history, each sample (1.2 to 1.8 mg) was heated from room temperature to 200°C and rapidly cooled down to room temperature, then the DSC scan was recorded by heating from 30 to 200°C. 

*X-Ray Diffraction Studies (XRD). *The diffractograms of the different samples were obtained by Philips PW 3040 equipment. The measurements were carried out at conditions: Ni-filtered CuK*α* radiation, voltage 50 kV, current 30 mA, scanning speed 1° (2*θ*)/min, and investigating the samples in the 2*θ* range 0–30°.

#### 2.2.5. *In Vitro* Dissolution Properties of Oxatomide-Cyclodextrin Complexes

The dissolution studies of oxatomide powder and the prepared oxatomide *β*-cyclodextrin solid complexes were performed according to the USP XXIII rotating basket method. The samples corresponding to 30 mg of oxatomide were placed into rotating basket. Dissolution medium was 900 mL of distilled water (pH 6.8). The stirring speed was 50 rpm, and the temperature was maintained at 37 ± 0.5°C. At predetermined times intervals samples of 5 mL were withdrawn by using syringe filter (0.45 *μ*m Millipore filter) and analyzed spectrophotometrically for oxatomide concentration at *λ*_max_ 277 nm. The withdrawn samples were replaced by equal volume of fresh dissolution medium. Each test was performed in triplicate.

#### 2.2.6. Bioavailability Study

The study was performed to assess the bioavailability of the suggested formula (oxatomide-*β*-cyclodextrin-PVPK15) selected on the basis of the dissolution studies in comparison with the reference oxatomide commercial product (Tinset 30 mg tablets). 

*(1) Study Design. *The study was approved by the Animal Ethics Committee of Faculty of Pharmacy, Helwan University. Guidelines of the ethics committee were followed for the study. Sixteen New Zealand white male rabbits (15 weeks old and weight 2.5–3 kg) were used in the study. All rabbits were housed and received similar diet. The rabbits were divided randomly into two groups; each was of eight rabbits and all rabbits were fasted overnight for 12 hrs with free access to water. On the day of experiment, each group received a single oral dose equivalent to 5 mg/kg oxatomide from each formulation by intragastric feeding tube. The doses of both products were suspended in a solution of 4.6% glycerin, 87.6% polyethylene glycol 400, and 7.8% distilled water.

The blood samples were withdrawn from central ear artery 1 hr before the drug administration and after administration at specific time points (0.5, 1, 2, 4, 6, 8, 10, 12, 24, 36, and 48 hrs). Blood samples were collected in heparinized tubes. The blood samples were centrifuged at 3,000 rpm for 10 min, and the plasma was transferred to separate glass tubes to be kept frozen until analysis. 

*(2) Analysis of Plasma Levels of Oxatomide*

*Oxatomide Sample Preparation.* Prior to analysis of plasma samples, aliquots of plasma (0.5 mL) spiked with 75 ng/mL flunarizine hydrochloride (internal standard) were vortexed for two min with (100 *μ*L × 1 M) sodium hydroxide solution (100 *μ*L × 1 M) and 5.0 mL ethyl ether. The samples were centrifuged at 2000 rpm for 10 min at room temperature. The upper organic layer was transferred to another centrifuge tube and evaporated to dryness under a gentle stream of nitrogen gas in a water bath at 40°C. The residues were then redissolved in 200 *μ*L mobile phase under vortex and centrifuged at 18000 rpm for 8 min. Aliquots of 5 *μ*L of the supernatant were injected into the liquid chromatography—mass spectrometry (LC-MS system). 

*LC-MS System. *Plasma samples were analyzed for oxatomide concentration by using a validated LC-MS assay [16] with some modifications. Briefly, LC-MS analysis was performed on an Agilent 1100 series LC/MSD chromatographic system (Agilent, USA) consisting of a water HPLC system equipped with an on-line solvent degasser, binary solvent delivery system, autosampler, and an Agilent technologies single quadrupole mass spectrometer with an electrospray ionization (ESI) interface. The MS detector has electrospray capability with a mass range of *m*/*z* 50–3000 and a mass accuracy of 0.1 amu. A liquid chromatographic separation was achieved on a Phenomenex C18 (250 × 2.0 mm i.d.) column, which was maintained at 40°C. The mobile phase consisting of 85% methanol and 15% (v/v) aqueous ammonium acetate solution (10 mM, pH 4.0) was pumped at an isocratic flow rate of 0.2 mL/min. The total run time was 7.5 min for each injection. 

The ESI source was set at positive ionization mode. The [M+H]^+^, *m*/*z* 427.10, for oxatomide and [M+H]^+^, *m*/*z* 405.05, for flunarizine hydrochloride were selected as detecting ions, respectively. The MS operating conditions were optimized as follows: nebulizer gas rate, 1.5 L/min; CDL temperature, 250°C; block temperature, 200°C; probe voltage, +4.5 kV. The quantification was performed via peak-area ratio. The lower limit of quantification was 5 ng/mL. The standard calibration curve for oxatomide was linear (correlation coefficients were >0.9975) over the studied concentration range (5–200 ng/mL). Data acquisition and processing were accomplished using Envirolab version 5 for the LC-MS system. 

*(3) Pharmacokinetic Analysis. *Oxatomide pharmacokinetics parameters were determined by noncompartmental kinetics [17]. The elimination rate constant (*K*_el_) was estimated by least square regression of plasma concentration-time data points in the terminal log-linear region of the curves. Half life (*t*_1/2_) was calculated as 0.693 divided by *K*_el_. The area under the plasma concentration-time curve from zero to the last measurable plasma concentration at time *t* (AUC_0−*t*_) was calculated using linear trapezoidal rule. The area under the curve from zero to infinity, AUC_0–*∞*_, was calculated as AUC_0–*∞*_ = (AUC_0−*t*_) + *C*_*t*_/*K*_el_, where *C*_*t*_ is the last measured concentration at the time *t*. Peak plasma concentration (*C*_max_) and the time to peak concentration (*T*_max_) were obtained directly from the individual plasma concentration versus time curve. 

#### 2.2.7. Statistical Analysis

In order to compare the results Student's *t*-test (SPSS program; version 12.0) was used. Data reported as mean ± standard deviation (SD). A statistically significant difference was considered at *P* value < 0.05.

## 3. Results and Discussion

### 3.1. Oxatomide-Cyclodextrin Phase Solubility Diagrams

**Figure 1:**
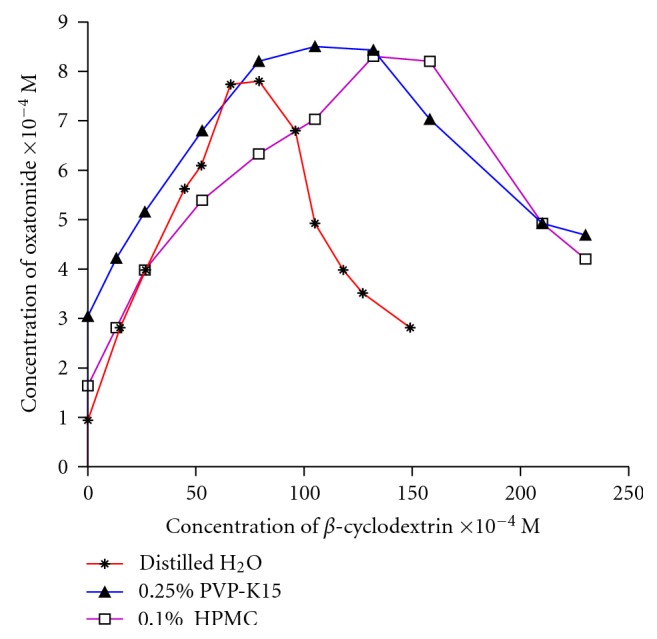
Phase solubility diagrams of oxatomide in different concentrations of *β*-cyclodextrin in distilled water and in presence of 0.1% HPMC, 0.25% PVP-K15. Each point represents the mean of measurements from three samples.

The phase solubility studies for oxatomide with *β*-cyclodextrin were carried out according to the method described by Higuchi and Connors [18]. The phase solubility diagrams of oxatomide in different concentration of *β*-cyclodextrin in distilled water, 0.1% HPMC solution, and 0.25% PVP solution are presented in Figure 1. The phase solubility profile of oxatomide *β*-cyclodextrin complex showed a typical Bs-type solubility curve, where the initial ascending portion is followed by a plateau region and then a decrease in total oxatomide solubility accompanied by precipitation of a microcrystalline complex [18].

The extent of complexation in aqueous media is characterized by the stability constant *K*_*s*_. The values of *K*_*s*_ were calculated according to the equation of Higuchi and Connors and from the initial straight line portion of the solubility diagrams by assuming that a 1 : 1 complex was initially formed according to the following equation:

(1) $$\begin{array}{c} K_{s}=\text{Slope}/\left[S_{0}\left(1-\text{Slope}\right)\right], \end{array}$$

where *K*_*s*_ is the stability constant for the complex and *S*_0_ is the solubility of oxatomide in absence of *β*-cyclodextrin.

The *K*_*s*_ value of oxatomide *β*-cyclodextrin inclusion complex was found to be [1443 M^−1^] in water and increased to [2027 M^−1^] and [1388 M^−1^] in presence of PVP-K15 and HPMC, respectively. 

### 3.2. Characterization of Oxatomide-Cyclodextrin Complex

#### 3.2.1. IR Spectroscopy

**Figure 2:**
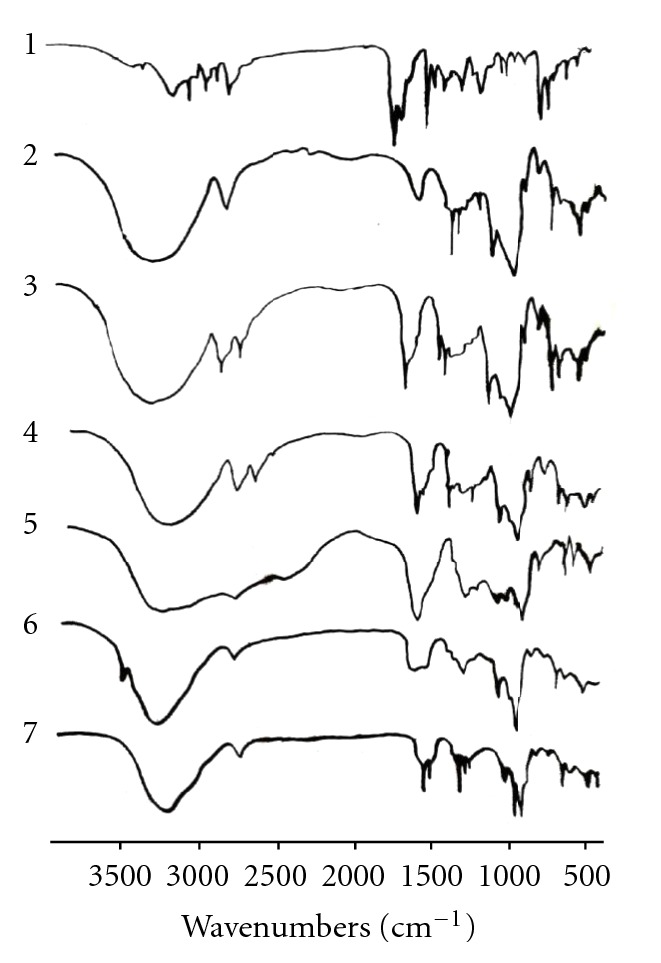
Infrared spectra of oxatomide (1), *β*-cyclodextrin (2), physical mixture (3), kneaded mixture (4), coevaporated (5), freeze-dried (6), and spray-dried (7) inclusion complexes of equimolar ratio of oxatomide and *β*-cyclodextrin.

IR spectra of oxatomide and its inclusion complexes with *β*-cyclodextrin prepared by different methods are presented in Figure 2. Oxatomide is characterized by the absorption of the carbonyl (C=O) group at 1702 cm^−1^; in the spectra of the inclusion complex, this band was shifted towards higher frequencies at 1727 cm^−1^ in case of coevaporated solid complex and to lower frequencies at 1682 cm^−1^ in spray-dried solid complex and decreased in intensity in freeze-dried complex. The absorption of N–H group in oxatomide at 3172 cm^−1^ was shifted toward lower frequencies at 2936–2924 cm^−1^ in all types of solid complexes. As spectral changes always concern C–OH, –CH_2_, and CH groups of the *β*-CD, it should be suggested that the host-guest interactions are dominated by hydrogen bonds among the above-mentioned groups.

#### 3.2.2. DSC Studies

**Figure 3:**
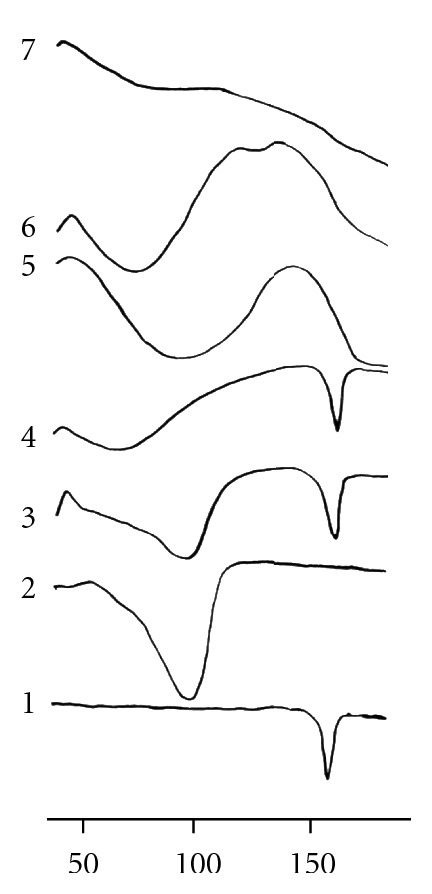
Differential scanning calorimetric thermograms of oxatomide (1), *β*-cyclodextrin (2), physical mixture (3), kneaded mixture (4), coevaporated (5), freeze-dried (6), and spray-dried (7) inclusion complexes of equimolar ratio of oxatomide and *β*-cyclodextrin.

The thermal behavior of oxatomide *β*-cyclodextrin inclusion complexes was studied using DSC to confirm the formation of the solid complexes. When guest molecules are incorporated in the cyclodextrin cavity or in the crystal lattice, their melting, boiling, and sublimation points usually shift to a different temperature or disappear within the temperature range at which the cyclodextrin lattice is decomposed [19]. The DSC curves for all the complexes are represented in Figure 3. The DSC thermogram of oxatomide exhibited one endothermic peak at 159.95°C. The DSC thermogram of *β*-cyclodextrin shows a very broad endothermic peak around 95°C corresponding to the release of water molecules. The thermogram of the physical mixture showed the melting endothermic peak characteristic of pure oxatomide and the broad peak corresponding to the dehydration of *β*-CD as if this thermogram was the superposition of those components, indicating the absence of interaction between oxatomide and *β*-cyclodextrin in such mixture. 

Thermograms of oxatomide *β*-cyclodextrin complexes prepared by kneading displayed a broad endothermic at 71.80°C due to the dehydration of the complex, and the peak at 160°C can still reflect the presence of a few drug crystals in the preparation. However, this thermal effect appeared more broadened and reduced in intensity, which suggests some drug-cyclodextrin interaction [20]. In addition to changes in the broad peak of *β*-cyclodextrin dehydration provided a further indication of the existence of inclusion complexes in these systems. However, the kneading and physical methods do not provide complete inclusion, and oxatomide was dispersed in the free state between inclusion complexes. The complete disappearance of the endothermic peak of pure oxatomide was observed in thermograms of freeze-dried, spray-dried, and coevaporated complexes and is attributed to the formation of an amorphous solid product or inclusion of the drug inside the *β*-cyclodextrin cavity or both [21]. 

#### 3.2.3. X-Ray Diffraction

**Figure 4:**
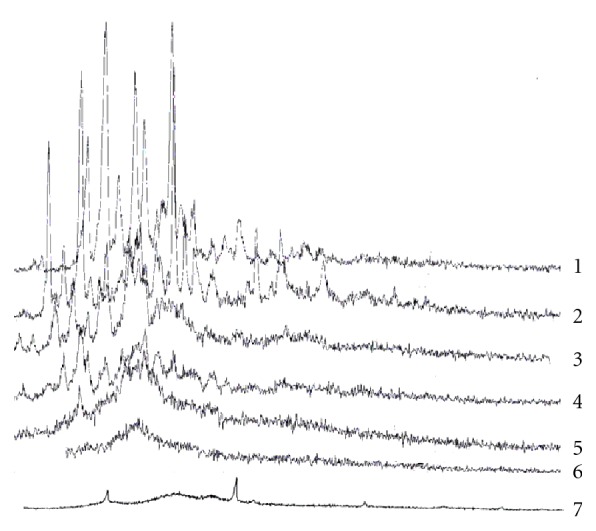
X-ray diffraction patterns of oxatomide (1), *β*-cyclodextrin (2), physical mixture (3), kneaded mixture (4), coevaporated (5), freeze-dried (6), and spray-dried (7) inclusion complexes of equimolar ratio of oxatomide and *β*-cyclodextrin.

The X-ray diffraction patterns of pure oxatomide, *β*-cyclodextrin, and their physical and kneaded mixture, coevaporated, freeze-dried, and spray-dried inclusion complexes are represented in Figure 4. The diffractograms of oxatomide and *β*-cyclodextrin exhibited a series of intense peaks which are indicative of their crystallinity. 

The differences in the diffraction patterns of physical mixture, coevaporated, kneaded, freeze-dried, and spray-dried complexes from each isolated constituent seem to indicate the formation of a new solid phase as a result of formation of inclusion complexes which is in good agreement with DSC studies. Furthermore, a reduction in peak height in diffraction pattern of the binary systems that was observed in all complexes indicated also a reduction in crystallinity. 

### 3.3. Dissolution Properties of Oxatomide-Cyclodextrin Complex

**Figure 5:**
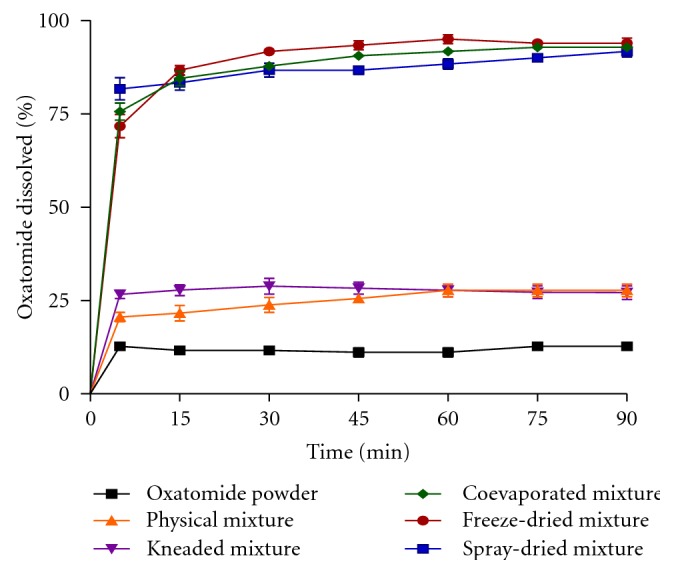
Dissolution profiles of oxatomide powder and corresponding *β*-cyclodextrin inclusion complexes in distilled water (pH 6.8).

As illustrated in Figure 5, it is clear that all inclusion complexes prepared by coevaporation, spray drying, and freeze drying exhibited higher dissolution characteristics than kneaded mixture, physical mixture, and pure oxatomide in distilled water.

The percentages of drug release after 5 min (*Q*_5_%), 90 min (*Q*_90_%), % dissolution efficiency (%DE) at 5 min (%DE_5 min_), and 60 min (%DE_60 min_) were determined and presented in Table 1. The %DE was calculated as the percent ratio of area under the dissolution curve up to time *t* (5 and 60 min), to that of the area of the rectangle described by 100% dissolution at the same time [22].

**Figure 6:**
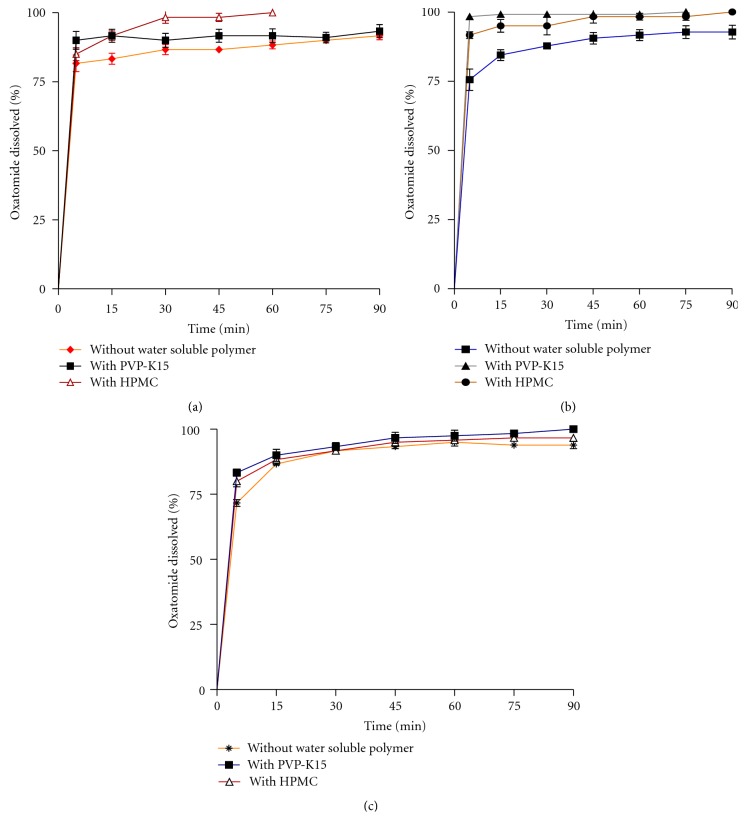
Dissolution profiles of spray dried (a), coevaporated (b), and freeze-dried (c) oxatomide *β* cyclodextrin inclusion complexes prepared in with or without water soluble polymer in distilled water (pH 6.8).

Regarding *Q*_5_, the inclusion complexes prepared by spray drying, freeze drying, and coevaporation (Figure 5) showed a prompt drug release compared to pure oxatomide (*P* < 0.05). The complex prepared by coevaporation in presence of PVP-K15 showed the highest release with *Q*_5_% and *Q*_90_% values of 98.33 ± 1.02% and 100%, respectively (Table 1 and Figure 6(b)). All inclusion complexes significantly increased the %DE_5 min_ and %DE_60 min_ (*P* < 0.05) compared to the pure drug (Table 1). Also, the complex prepared by coevaporation in presence of PVP-K15 showed the highest %DE. The increase in dissolution of drug in presence of both *β*-cyclodextrin and water soluble polymers may be attributed to both improvement of drug wettability and formation of readily soluble complexes in dissolution medium. When oxatomide *β*-cyclodextrin inclusion complexes come in contact with an aqueous dissolution medium, the hydrophilic carrier dissolves and results in precipitation of the embedded drug into fine particles, which increase the dissolution surface available [23]. 

### 3.4. Bioavailability Study

**Figure 7:**
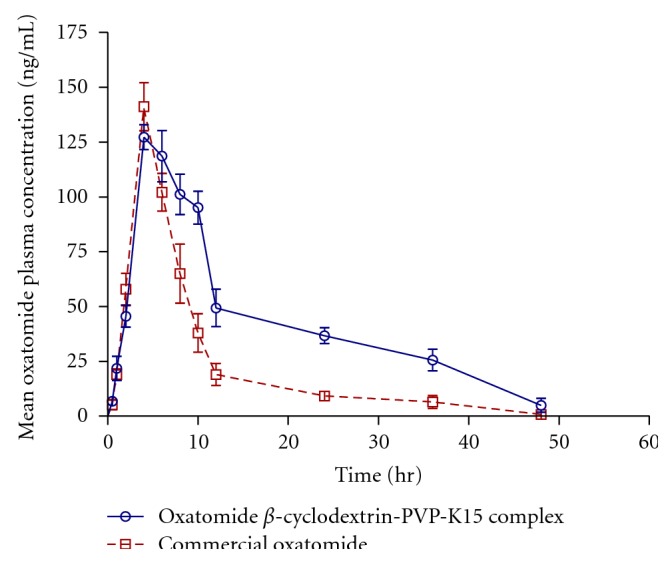
Mean plasma concentration—time curves of oxatomide in rabbits (*n* = 8) after administration of a single oral dose (5 mg/kg) of oxatomide-*β*-cyclodextrin-PVPK15 complex and commercial oxatomide product.

The mean plasma oxatomide concentration-time profile following administration of single oral dose of oxatomide-*β*-cyclodextrin-PVPK15 complex and the commercial drug product (Tinset) are shown in Figure 7. Pharmacokinetic parameters (*C*_max_, *T*_max_, AUC_0–48_ and AUC_0–*∞*_) are calculated individually on the basis of concentration-time data. From individual pharmacokinetic parameters, their mean values ± S.D, were obtained and are shown in Table 2 for both formulations. 

Statistical analysis of the bioavailability parameters AUC_0–48_, AUC_0–*∞*_, and *C*_max_, data obtained for oxatomide-*β*-cyclodextrin-PVPK15 complex showed significantly (*P* < 0.05) higher values of AUC_0–48_ and AUC_0–*∞*_ and significantly lower values of *C*_max_ compared to commercial oxatomide product. The mean *T*_max_ values for oxatomide-*β*-cyclodextrin-PVPK15 complex formulation and the commercial oxatomide product were 4.5 ± 0.92 and 4 ± 0.00 hrs, respectively; the difference between the mean *T*_max_ values was found to be statistically insignificant (*P* > 0.05) when analyzed Student's *t*-test. 

The individual AUC_0–*∞*_ values for oxatomide-*β*-cyclodextrin-PVPK15 complex were compared to those for the commercial product to determine the relative bioavailability. The mean relative bioavailability of oxatomide-*β*-cyclodextrin-PVPK15 complex to the commercial product was 173.15 ± 16.53 %. This result indicated that 73.15% increase in the oral bioavailability of oxatomide was achieved by the complex formulation. Although oxatomide *β*-cyclodextrin complex improvesthe bioavailability of oxatomide in terms of extent of absorption from the GI tract, it did not change the rate of drug absorption in terms of *T*_max_ and achieved significantly lower *C*_max_ values in comparison with the commercial drug product. Comparing the values of elimination rat constants (*K*_el_) for oxatomide-*β*-cyclodextrin-PVPK15 complex with those for commercial product reveals a marked effect of complexation on increasing the duration of action of oxatomide.

## 4. Conclusion

The results reported here suggest that inclusion complex of oxatomide with *β*-cyclodextrin prepared by coevaporation in presence of PVP-K15 results not only is an enhancement of the oxatomide dissolution rate but also improvesthe bioavailability of oxatomide in terms of extent of absorption from the GI tract and increases the duration of action of oxatomide.

## Figures and Tables

**Table 1:** The percentage of drug release after 5 min (*Q*_5_%), 90 min (*Q*_90_%), and % dissolution efficiency (%DE) at 5 min (%DE_5  min_) and 60 min (%DE_60  min_) of oxatomide from different inclusion complexes in distilled water (pH 6.8 ) at 37°C ± 0.5°C.

Formulation	Dissolution parameters			
	*Q*_5_%	*Q*_90_%	%DE_5 min_	%DE_60 min_
Oxatomide powder	12.70 ± 0.23	12.70 ± 1.00	6.35	11.07
Physical mixture	20.55 ± 1.22	27.70 ± 1.67	10.27	22.85
Kneaded mixture	26.60 ± 1.10	27.10 ± 1.80	13.30	26.84
Coevaporated mixture	75.57 ± 2.26	92.78 ± 2.50	37.77	83.07
Coevaporated in presence of PVP-K15	98.33 ± 1.02	100 ± 0.00	49.16	94.93
Coevaporated in presence of HPMC	91.67 ± 1.20	100 ± 0.00	45.83	91.87
Spray-dried mixture	81.67 ± 2.98	91.67 ± 1.45	40.83	81.94
Spray-dried in presence of PVP-K15	90.00 ± 3.21	93.33 ± 2.34	45.00	87.00
Spray-dried in presence of HPMC	85.00 ± 2.33	100 ± 0.50	42.50	91.38
Freeze-dried mixture	71.67 ± 3.10	93.88 ± 1.40	35.85	85.14
Freeze-dried in presence of PVP-K15	83.33 ± 1.34	100 ± 0.00	41.66	88.85
Freeze-dried in presence of HPMC	80.00 ± 2.10	96.67 ± 1.23	40.00	87.04

**Table 2:** Pharmacokinetic parameters of oxatomide in rabbits (*n* = 8) after administration of a single oral dose (5 mg/kg) of oxatomide-*β*-cyclodextrin-PVPK15 complex and commercial oxatomide product.

Parameter	Oxatomide-*β*-cyclodextrin-PVPK15 complex	Commercial oxatomide product
*C*_max_ (ng/mL)∗	127.23 ± 6.180	141.16 ± 10.99
*T*_max_ (hr)	4.50 ± 0.925	4.00 ± 0.00
AUC_0–48_ (ng·hr/mL)∗	1586.90 ± 84.00	957.10 ± 57.00
AUC_0–∞ _(ng·hr/mL)∗	1662.14 ± 127.06	962.85 ± 57.61
*K*_el_, (hr^−1^)∗	0.074 ± 0.016	0.133 ± 0.028
Relative bioavailability (%)	173.15 ± 16.53	—

Each value represents the mean ± standard division of eight rabbits.

∗ *P* < 0.05 based on Student's *t*-test, statistically significant difference between oxatomide-*β*-cyclodextrin-PVPK15 complex and commercial oxatomide product.
